# Assessing the Importance of Health in Choosing a Restaurant: An Empirical Study from Romania

**DOI:** 10.3390/ijerph16122224

**Published:** 2019-06-24

**Authors:** Gabriela O. Chiciudean, Rezhen Harun, Iulia C. Muresan, Felix H. Arion, Daniel I. Chiciudean, Garofita L. Ilies, Diana E. Dumitras

**Affiliations:** 1 Department of Economic Sciences, University of Agricultural Sciences and Veterinary Medicine Cluj-Napoca, 3-5 Manastur Street, 400372 Cluj-Napoca, Romania; gabriela.chiciudean@usamvcluj.ro (G.O.C.); iulia.muresan@usamvcluj.ro (I.C.M.); felixarion@usamvcluj.ro (F.H.A.); daniel.chiciudean@usamvcluj.ro (D.I.C.); garofita.ilies@usamvcluj.ro (G.L.I.); 2 Department of Agribusiness and Rural Development, College of Agricultural Sciences Engineering, University of Sulaimani, Kurdistan Regional Government-Iraq, Sulaimani- Bakrajo, Sulaimani-IRAQ 334, Iraq; rezhen.rashid@univsul.edu.iq

**Keywords:** consumer choice, restaurant, healthy meals

## Abstract

In the context of a spectacular growth of the Romanian restaurant market, it has become a necessity for managers to analyze the decision-making process related to restaurant selection toward obtaining a competitive advantage, which can be achieved through better segmentation and adequate targeting. The main objectives involved the identification of the main factors that influence restaurant selection by evaluating the role that health concerns play in this process and identifying consumers’ restaurant profiles. A survey was conducted using face-to-face interviews as the contact method, in order to identify the main factors considered important for consumers in the city of Cluj-Napoca in the decision-making process related to restaurant selection. Principal component analysis (PCA) was conducted to group the attributes. The non-hierarchical cluster analysis through the use of the k-means method was used to define different groups within the sample and identify common features. Results suggest that the analyzed restaurant market is dominated by three segments of consumers, of which the largest is represented by “health seekers”: a group of young women with medium and low incomes. As such, the possibility of consuming healthy meals within a restaurant is the most important factor for them during the decision-making process for restaurant selection. The present study has important managerial implications. Restaurant managers should admit that this process represents the starting point in designing restaurant concepts, as this type of information is fundamental for management decisions. On the other hand, the study offers important information regarding consumer perception of food, which has recently changed significantly, especially in the segment of young consumers to whom health is very important.

## 1. Introduction

According to the Euromonitor International report [1], the market of Romanian restaurants has grown in a spectacular way since 2017 as a result of the increase of minimum wage, salaries, and pensions, enabling consumers to dine more often at restaurants. Also, the segment of full-service restaurants increased substantially as a consequence of the increasing popularity of different niches such as vegans, vegetarians, etc. The total market value of restaurants in five of the largest cities in Romania (Bucharest, Cluj-Napoca, Timisoara, Brasov, and Iasi) reached 7.24 billion lei (1.54 billion Euro) in 2018 [2]. The Horeca market for Cluj county is the second most dynamic in Romania, following Bucharest, the capital city, with a large number of new units addressing different market segments and niches. The rapid growth is due also to some city strengths developed over the years such as the title of “European Youth Capital in 2015”, as well as the new nickname “Silicon Valley of Romania”, the academic center, the Transylvania International Film Festival, and the business center [3]. In this context of growing competition, identifying the most important selection attributes within the decision-making process of dining out represents a crucial step [4] in building adequate marketing strategies based on a better segmentation and an efficient targeting [5,6], thus contributing to customer satisfaction.

Previous studies have mainly focused on the selection attributes and the factors that influence consumer decisions regarding dining out. It has been observed that generally, the most important influence factors are related to “food quality” and “service quality”, while factors related to “marketing and promotion” or even “price” are not so important [4,5,6,7,8,9]. 

Most researchers focused on identifying the factors that influence consumer choice regarding restaurant selection in order to understand the starting point in building marketing strategies [10,11,12,13,14,15,16,17,18,19,20,21] but few of them clustered the consumers based on the influencing factors [9,22,23]. In Romania, there is a lack of such studies describing consumer segments based on factors that affect their choice of dining in restaurants. Strategies based on market realities can better support management decisions and respond to unexpected market changes with less impact on business development for the long term [24]. As such, this study aims to provide insights into this important issue of understanding consumer choices when dining out. The main objectives set are: (1) identifying the main factors that influence the selection of a restaurant by evaluating the role that health concerns play in this process; and (2) identifying consumers’ restaurant profiles.

The paper is structured into six main sections. The introduction is followed by a section related to a literature review of the factors influencing the decision-making process of restaurant selection, along with the market segmentation of restaurants and the main socio-demographic characteristics that influence restaurant selection. The Section 3 presents the research area and describes the research methodology. Furthermore, the results are presented in the Section 4, while the Section 5 is dedicated to the discussion. The paper ends with the conclusions and implications of the study, which are presented in the Section 6.

## 2. Literature Review

Provided that there is strenous competition in the restaurant business, Yi et al. [25] considered there to be two issues essential for successful restaurant management: knowing the main characteristics of the restaurant target market, and understanding the process of restaurants’ selection in building competitive factors. The degree of importance given to these selection factors when choosing a restaurant is directly related to food service, dining context, and the consumers’ socio-demographic profiles [26,27].

### 2.1. Factors Influencing Restaurant Selection

The importance of awareness and understanding of the main factors that affect consumer choice of sit-down restaurants is emphasized by Jalisa et al. [28], who identified two main categories of factors: internal features (menu, staff, food, atmosphere, etc.) and external features (location, competitors, economic status, etc.).

Among the internal features, food quality is considered a crucial factor of influence in the selection process of a restaurant [10,11,12,13,29,30,31,32] and a predictor of customer satisfaction [14,33,34]. It consists of two dimensions: organoleptic and presentation [12]. However, a study conducted on full service restaurants highlights that customers have greater expectations that exceed food quality by focusing on the quality of the service [15]. The type of restaurant could influence consumer hierarchy and sometimes places “food quality” on a lower rung, as in the case of an upscale ethnic restaurant where “experience” was found to be the most important factor of choice [17] or as important as food quality [18]. Similar results were obtained by Basri et al. [35] analyzing a Malaysian upscale restaurant, where food quality was exceeded in importance by the physical environment. Hygiene factors were identified by some scholars as being more important than food [36]. 

The perceived overall quality of a restaurant (referring to quality of service, quick service, and friendliness of staff) represents another important factor of influence for consumers, and is often critical in the phase of selection, over the price and menu variety [13,15,37]. Kattara et al. [38] mentioned the existence of a direct correlation between the overall perceived quality of a restaurant and the employees’ behavior (positive or negative). Service quality proved to be the most important factor determining the perceived negative experience related to a restaurant, exceeding the quality of food or drinks, the friendliness of staff, and the cleanliness of the space [20]. It is worth mentioning that in the case of travelers, cleanliness was more important than food or friendliness of staff [39], ranking this factor in the top three restaurant selection factors [40]. It is not only the cleanliness of spaces that matters, but also the safety of food handling and preparation, which are often invisible to the consumers, and became a serious issue of public health concern in the canteens of Vietnam [41]. 

The price factor has many particularities that affect consumer behavior in the process of restaurant selection. Analysis of the consumers’ willingness to pay for three major attributes such as “food quality”, “service”, and “ambiance”, allowed a clear distinction between the types of restaurants. That is, consumers were willing to pay a higher price for food quality in upscale restaurants, while in the case of fast-food restaurants, the service quality and especially the speed of service represented the most important influence factor that consumers are more willing to pay for [10,11]. In the case of fine dining restaurants, price was the least important attribute, while customer relations proved to be the most important [42]. 

When considering external features, but strictly those related to food quality, the nutritional concern is worth mentioning, as it is gradually on the rise [19] with major implications in the restaurant industry. The inclusion of healthy food in menus has recently become an important factor of choice [8], and the dietary considerations have a definite impact on the decision of selecting a dining place [7,21]. It was observed that lifestyle (weight concern, being on a diet, and knowledge about the food pyramid) is an important factor of influence when choosing healthy food; therefore, the service quality and the existence of healthy food in the menu has a positive influence on the intention to return [8]. Generally, the lack of knowledge about food characteristics and the unfamiliarity with a type a food generates fear and avoidance among consumers [43]. Furthermore, online reviews and restaurant ratings have become as important as the perceived food quality in the decision-making process of selection [16].

It was observed that in recent years, the importance of price was exceeded by brand image for consumers in the United States (USA), asserting its influence on all the attributes in the decision-making process for choosing a restaurant [25]. 

Starting from the classification made by Jalisa et al. [27], an inventory was conducted by grouping all the factors of influence identified in the above-mentioned studies into internal and external features (Table 1).

It is also argued that restaurant managers should consider these factors starting with the phase of restaurant concept creation, as the wrong one is often found to be the main cause of failure [26]. Even though the selection attributes that are considered to be important for consumers when dining out represented an area of research in other countries [10,11,12,13,14,15,16,17,18,19,20,21,22,23,24,25,26,27,28,29,30,31,32,33,34,35,36,37,38,39,40,41,42,43], no similar studies were identified in Romania.

### 2.2. Market Segmentation of Restaurant Consumers

Market segmentation is essential for the restaurant industry, as it may be considered to be the cornerstone of marketing management [23]. Therefore, scholars focused on identifying segments of consumers based on the factors that affect the decision-making process of restaurant selection [9,22,23]. Indian consumers were grouped into three clusters: “deal seekers” look for facilities, service quality, tasty food, ambiance, and cleanliness; “preference seekers” appreciate a personalized experience, where hygiene is more important than taste and children facilities are also required; and “experience seekers” appreciate the overall experience of a restaurant, without any concern for taste of food or hygiene [9]. The German and English consumers were divided into five clusters: “value seekers” select restaurants that offer food value for money, food quality, hygiene, and visibility of food preparation; “service seekers” want quality services and secondly, food quality; “adventurous-food seekers” look for interesting, local food; “atmosphere seekers” mainly search for pleasant atmosphere and a good time; “healthy-food seekers” search for those restaurants with quality healthy food and hygiene [22]. The segment of fine-dining Malaysians is divided into five clusters, which are based on their personality patterns: “laid-back consumers” are represented by passive, careless consumers; “prudent consumers” manage any situation carefully; “objective consumers” base their decisions on factors rather than impulse; “cautious consumers” are attentive to special prices and promotions; and “loyalists” have one preferred restaurant [23].

Scholars have paid strong attention to the “generation Y” consumers [20,44], analyzing closely their behavior and attitudes toward food. Njite et al. [42] identified four segments of consumers by means of cluster analysis based on the factors considered important when selecting a restaurant: the “adventurous consumers” (who considered “value and service reliability”, “restaurant reputation”, and “food quality” as the most important); the “convenience-oriented consumers” (the lowest mean score for “natural/organic ingredient” and “nutritional/healthy menu”); the “health-conscious consumer” (the highest mean scores for “natural/organic ingredient” and “nutritional/healthy menu”), and the “unconcerned consumer” (with disregard for of the attributes). Based on researcher knowledge, in Romania, there are no similar studies that group consumers into distinctive segments based on the factors influencing the decision to select restaurants according to consumer socio-demographic characteristics. 

### 2.3. The Influence of Socio-Demographic Characteristics on the Process of Restaurant Selection

Consumer socio-demographic characteristics influence the process of restaurant selection by focusing on particular attributes. For instance, generation Y (people born between 1980–2000) represents an important demographic group that must be taken seriously into consideration by restaurant managers, as they are frequent consumers with the capacity to influence their friends and family [20,44]. Their expectations regarding dining differ from other segments, placing the quality of the service (speed, friendliness of staff, cleanliness) in the first position, followed by the quality of food and the ambiance [20]. Higher age groups and higher budget groups considered the visual presentation of food and ambiance as essential factors in differentiating restaurants from one another [45]. On the contrary, in the case of the lower-income population, the price was found to be the most important attribute. In Hong Kong, differences were observed in consumers aged 45 and above, which was the only segment interested in facilities for children [13]. In terms of gender, it was observed that a higher importance was granted by women to safety and their family preferences when choosing a restaurant for dining [26]. Although the influence of socio-demographic characteristics is notable in previous studies conducted in other countries, similar research has not been conducted in Romania.

## 3. Materials and Methods

A survey was conducted from January to June 2017 using face-to-face interviews as the contact method, in order to identify the main factors considered important for consumers in Cluj-Napoca city during the decision-making process of restaurant selection. Responses were collected from 276 consumers using the convenience sampling approach. Respondents were recruited in the proximity of restaurants at predetermined locations in Cluj-Napoca (Museum Square, Heroes Street), where consumers can find a large variety of restaurants due to the difficulty of obtaining permission to approach customers while dining inside the restaurants. Similar methods were used in previous research [7,22,46]. The sample size met the subject-to-item ratio of at least five items per subject, which is the minimum recommendation in exploratory factor analysis [47,48]. The questionnaire consisted of 24 questions divided into three sections: the consumer behavior of restaurants, the attributes considered important in the decision-making process of restaurant selection, and the socio-demographic profile of the respondents. The attributes included in the second section were adapted from previous studies [4,5,7,16,20,22] and evaluated on a scale from 1 to 5, where 1 meant totally unimportant, while 5 meant very important. There was no general consensus among other researches regarding the attributes that must be analyzed, therefore, they differ from one research to another. From the initial 20-item list, three were excluded as they had high factor loading in more than one factor [49]. The eliminated items were “smoking area”, “quick service”, and “fresh products”. The third section of the survey instrument was used to design the consumer socio-demographic profile. The respondents needed between 12–15 min to fill in the questionnaire. 

Data were analyzed using an SPSS 20.0 software package (SPSS Inc., Chicago, IL, USA). Descriptive statistics was used to describe the socio-demographic profile of the consumers and indicate the means and standard deviations of each of the attributes considered important in the decision-making process of restaurant selection. Principal component analysis (PCA) was conducted to group the attributes (17 items measured using a 5-point Likert-style scale where 1 = totally unimportant, 2 = unimportant, 3 = neutral, 4 = important, and 5 = very important). This technique permits defining the latent factors that seem to influence restaurant selection. Varimax rotation was used to maximize the differences among the extracted components and maintain correlation among the components. The Kaiser–Meyer–Olkin (KMO) test of sampling adequacy and Bartlett’s test of sphericity were carried out to determine the fitness of the data. All the factors with eigenvalues higher than one were retained for further analysis [50]. Next, the non-hierarchical cluster analysis (k-means method) based on the regression factor scores identified in the PCA was used to define different groups within the sample. This procedure allows for grouping the consumers based on common features, thereby creating restaurant market segmentation based on consumer choices. PCA was performed before the clustering analysis [51] because of the dimensionality reduction capacity as a feature extractor and the possibility to visualize/reveal clusters and validate the clustering algorithm [52]. 

The number of clusters was determined based on a preliminary study of the dendrogram obtained by using the hierarchical method with Ward’s method. The Shapiro–Wilk test was used to test the normality of the statements (*p* < 0.05), and the Kruskal–Wallis test was used to compare the groups. Contingency table analysis and the logistic regression analysis were employed to identify the main socio-demographic characteristics of the groups.

## 4. Results

### 4.1. Socio-Demographic Profile of the Respondents

Table 1 shows that the sample is gender balanced. The young segments of respondents (18–25 years) are represented by almost half of respondents (39.9%), followed by the group aged 26–30 years, which represents 22.1% of the total sample. Respondents aged between 41–50 years old represented only 9.4% of the respondents, while elders (aged 50+) represented the smallest percentage of 3.3%. About 60% of respondents had small to medium incomes (up to 445 euros per month) (Table 2).

### 4.2. Factors Influencing Restaurant Selection

The PCA was conducted to explore the factors that may influence restaurant selection, by determining the dimensionality of the 17 items presented to respondents. The Bartlett test of sphericity was significant (Chi-square = 3609.931, *p* = 0.000), providing support for the validity of data. The Kaiser–Meyer–Olkin overall measure of sampling was 0.852, indicating that the data were appropriate for PCA [53]. The exploratory factor analysis with varimax rotation of the 17 variables resulted in a four-factor solution, accounting for 71.4% of the total variance. All four factors had eigenvalues greater than one. Cronbach’s alpha reliability coefficient was computed to establish the internal consistency of each component. The four underlying dimensions resulted from the PCA are presented in Table 3, while the average score assigned by the respondents to each item indicated the importance of the item in the selection of restaurants, and is reported in Table 4. The overall reliability of the 17 variables was 0.9, indicating a high level of homogeneity among items. 

The first dimension, named “Price quality, service, and marketing”, explained 39.86% of the total variance with a reliability coefficient of 0.93. It was comprised of seven attributes with a mean of 3.44 ± 0.158, suggesting that it is quite neutral from the point of view of the importance given by the respondents (Table 4). This factor referred to the attributes that combine a low price with good quality service (product presentation and rapidity of preparation), but also a good reputation (given by positive ratings in food guides, favorable reviews) and attractive look (interior design and special promotions). Among the attributes included, “rapidity of preparation” (mean = 3.70, SD = 1.173) and “product presentation” counted the most.

The second dimension entitled “Product quality and variety of the menu” comprised six attributes, which explained 17.17% of the total variance with a reliability coefficient of 0.87. This component had a mean of 3.65 ± 0.388. Among the attributes comprised by this dimension, the most important were related to the freshness of the ingredients (mean = 4.21 ± 1.156) and the variety of the menu (mean = 4.07, SD = 1.073). Three attributes relate to meal consistency: traditional food (mean = 3.47 ± 1.158), hearty meals (mean = 3.46 ± 1.090), and high nutritional value of food (mean = 3.43 ± 1.078), all with almost the same importance expressed by consumers when choosing a restaurant. The least important attribute is the presence of international meals (mean = 3.26 ± 1.047).

The third factor labeled “Healthy meals” contained two attributes, and it had a mean of 3.54 (mean = 3.54 ± 0.015). The 0.82 reliability coefficient explained 7.35% of the total variance. This factor comprised the attributes that refer to the importance of low-calorie meals (mean = 3.53 ± 0.978) and dietary meals (mean = 3.55 ± 1.170) when choosing a restaurant. 

Even if the “appearance” component had the highest degree of importance when choosing a restaurant (mean = 4.35 ± 0.368), it was decided to remove this factor from further investigations due to the commonality value below 0.6 (α = 0.54) (Table 4).

### 4.3. Consumer Segmentation Based on the Important Factors in the Decision-Making Process of Restaurant Selection

The non-hierarchical cluster analysis led to three homogeneous clusters identified based on the three dimensions, suggesting the presence of defined segments on the market. The identified clusters are: “Price quality, service, and marketing”, “Product quality and variety of the menu”, and “Healthy meals”. The final cluster centers are reported in Table 5, indicating the importance of the factors for each cluster. “Price quality, service, and marketing” was the factor with the highest influence on clustering the respondents, while the other two factors, “Product quality and variety of the menu” and “Healthy meals” had less influence. In addition, the Kruskal–Wallis test indicated a statistically significant difference between the mean values of the factors across the clusters (*p* < 0.001) (Table 6).

The clusters were labeled according to the factors considered to be distinctive for the respondents in the decision-making process of restaurant selection: Cluster 1, “Healthy-meal seekers” (*n* = 124); Cluster 2, “Laid-back consumers” (*n* = 43); Cluster 3, “Deal seekers” (*n* = 109). Statistically significant differences were found between the groups with respect to gender, age, marital status, and the personal monthly income of respondents (Table 7).

Cluster 1, “Healthy-meal seekers”, was the dominant group and represented 44.9% of the consumers. Cluster 1 seemed to have the highest mean score for the “Healthy meals” factor (mean = 4.37 ± 0.521), followed closely by the “Product quality and variety of the menu” factor (mean = 4.04 ± 0.633). The lowest mean score was registered for “Price quality, service, and marketing” (mean = 3.89 ± 0.561) (Table 6). Thus, it can be assumed that consumers from this group of respondents select restaurants according to the possibility of consuming healthy meals of high quality and variety, being less responsive to marketing efforts and price. The first group is quite balanced regarding the respondents’ gender, although female respondents have a slightly higher percentage (56.6%). As related to the age variable, it can be observed that it grouped a large percentage of young respondents (54.7% aged between 18–25 years and 19.8% aged between 26–30 years). Moreover, this cluster exhibited the highest share of unmarried people (83.0%) and a relatively high small and medium income (29.8% gain between 320–445 euros, while 33.3% were below 320 euros).

Cluster 2, “Laid-back consumers”, represented the smallest group, with only 15.57% of the respondents. It had a lower mean score on “Price quality, service, and marketing” (mean = 1.70 ± 0.495) and also on “Product quality and variety of the menu” (mean = 2.91 ± 1.062), suggesting that these aspects are not important in their choice. They also seem to be indifferent to “Healthy meals” (mean = 3.14 ± 0.786). The second cluster exhibited the highest share of female consumers (62.8%), and tended to be young consumers (56.1% aged between 18–30 years), but also with small and average income almost equally distributed below 555 euros. 

Cluster 3, “Deal seekers”, was the second largest group of consumers after the “Healthy-meal seekers”, representing 39.49% of the total sample. Cluster 3 had the lowest mean score for “Healthy meals”, which was unimportant to them (mean = 2.74 ± 0.629). Almost as important are the factors labeled “Price quality, service, and marketing” (mean = 3.62 ± 0.558) and “Product quality and variety of the menu” (mean = 3.48 ± 0.762). Regarding their age, the consumers from the third cluster are mostly young people (48.3% aged between 18–25 years), their income being medium to low (58.5% relying on less than 445 euros per month). 

By using logistic regression, it was possible to assess the extent to which gender, age, income, and marital status affected the chances of belonging to each identified cluster (Table 8). The dependent variable took a value of one if respondents belonged to the respective cluster. Income level and marital status were assessed as not significant, and were thus excluded from the final models. Being male reduced the chances of belonging to the “Healthy-meal seekers” cluster compared to being female (odds ratio = 0.522, *p* = 0.026). Chances of belonging to Cluster 1 were also reduced for people 41–50 years old as compared to young people 18–25 years old. With regard to Cluster 2, the chances of belonging to this group were reduced for male consumers (odds ratio = 0.467, *p* = 0.042). Higher chances of belonging to this group were found for people between 26–50 years old, which was a relatively wide range indicating that the indifference toward “healthy meals” can be found at various ages. On the other hand, being male increased the chances of belonging to the “Deal seekers” cluster (odds ratio = 3.145, *p* = 0.000). When compared to people 18–25 years old, the chances of belonging to this cluster were lower for people between 31–40 years (odds ratio = 0.212, *p* = 0.009). 

## 5. Discussion

The results indicated that the decision-making process of restaurant selection had some particularities for the respondents in Cluj-Napoca, which is a reality that should be considered key information for managers in developing marketing strategies for obtaining proper results and prevent failure, as suggested also by Jalisa et al. in their study [28]. The respondents in the categories of age between 18–30 years dominated the sample, which was in line with a study conducted at the national level on the consumption habits of Romanians, which underlines that young people are the most interested in dining out because of the need to socialize. According to the same study, the frequency of dining at a restaurant decreases with age; the percentage of the consumers aged over 34 was 55% smaller [54]. However, this is not an isolated phenomenon. Considered as the next important segment of spending [20,44], the Generation Y segment (aged between 16–34 years) has some major and important characteristics that are not always understood by researchers. This generation is strongly influenced by the internet and technology and has the ability to influence their friends in its turn; therefore, there is a general consensus that their behavior must be thoroughly analyzed [55,56,57,58,59] and related particularly to restaurants [20].

The analysis of the mean values for the attributes considered important in the decision-making process of restaurant selection indicated that the most important attribute for respondents is the “cleanliness of spaces”, which is a finding supported by other research [5,6,36,38,40,59,60,61,62], and is followed by food made from “fresh ingredients (not frozen)” [5,31]. Therefore, unlike other studies, which place “food quality” on the first rank when it comes to a restaurant selection (an intrinsic attribute) [10,11,12,13,14,29,30,31,32,33,34], Romanian consumers in Cluj-Napoca pay much more attention to “cleanliness of spaces” (an extrinsic attribute). Other important attributes were found to be the “ambiance” and the “variety of the menu”. Respondents remained mostly neutral to attributes such as the “interior design” and the “promotions”. Not even “ratings” were important to respondents; this was different from other research where restaurant ratings represented a main attribute [16,63,64] after food quality [16]. After conducting the PCA, the attributes were grouped into four dimensions, reinforcing previous studies [4], but only three of them met the necessary criteria (communality over 0.6): “Price quality, service, and marketing”, “Product quality and variety of the menu”, and “Healthy meals”.

The clustering of the respondents based on the factors considered important in the decision-making process of restaurant selection yielded three segments of consumers: “Healthy-meal seekers” was the dominant group and represented 44.9% of the consumers; “Laid-back consumers” represented the smaller group with only 15.6% of the respondents, and “Deal seekers” was the second largest group of consumers, representing 39.5% of the total sample. The distinctive socio-demographic characteristics were identified for all three segments of consumers.

The first cluster, which was labeled “Healthy-meal seekers” was in accordance with similar segments identified by other scholars [5,44]; for this demographic, the possibility of consuming healthy, dietary, fresh, qualitative, and varied meals is the most important issue in the process of restaurant selection. For this segment, aspects such as marketing efforts and price quality are neutral. Even if a relatively balanced structure regarding the respondents’ gender could be noticed, the female respondents have the highest percentage (55.6%); this finding is supported by a study conducted in 10 countries [65] which indicated that young women are more interested in healthy restaurant meals (fresh ingredients and vegetables) and, unlike men, they perceive healthy meals as tasty. Moreover, female consumers have a higher chance of belonging to the group of “Healthy-meal seekers”. The same scholars [65] mentioned that the preference for healthy food decreases with age, as in the case of the first identified segment, where the chances of belonging to this group are reduced as age increases. Although previous studies identified a strong relationship between low economic status and support for unhealthy food [66], highlighting that healthy meals are a priority even if financial resources are limited, in the current study, income was not found to be a distinctive characteristic of this group. Jin et al. [67] emphasized the importance for marketers to be aware of health considerations having been recently included in the process of decision-making when selecting a restaurant, and a main reason in some cases [68]. The consumers interviewed seem to have developed a strong health consciousness and practice healthy dietary habits, which are considered by scholars to be key factors in improving public health [69,70], as changing consumer food behavior is challenging [71]. The presence of an important segment of “health-conscious consumer” was observed by Jang et al. [44]. This type of consumers considers health to be a priority, deeply analyzes the product information, and is willing to pay a premium for nutritional and natural products. 

The second cluster, “Laid-back consumers”, is labeled—as in the case of Malaysian consumers [23]—similar to the “unconcerned consumers” [44]. It is the smallest group of respondents of mostly females with small or average income, half of them being aged below 30 years, and almost 25% between 40–50 years. The chance of belonging to this cluster depends on gender and age, and are higher for female consumers and consumers between 26–50 years old. Their main characteristics are similar to the ones described by Talib et al. [23], namely passive, careless consumers, with disregard for all of these factors. 

The third cluster labeled “Deal seekers” was the second largest group of consumers following the “Healthy-meal seekers”, representing 39.5% of the total sample. To this group, “Healthy meals” is an unimportant aspect when selecting a restaurant, but they pay some attention to “Price quality, service, and marketing” and the “Product quality and variety of the menu”. This segment comprises mostly young people with relatively low income. The chance of belonging to this cluster is affected by gender and age, and is higher for male consumers and younger consumers. Labeling was based on the similar segment identified among Indian consumers who consider facilities, service quality, tasty food, ambiance, and cleanliness as important factors [9]. The segment is similar to the “convenience-oriented consumers” identified by Jang et al. [44]. 

## 6. Conclusions

The present article aimed to identify the main factors that are considered important by consumers in the decision-making process of selection for sit-down restaurants, which is information that is recognized as important for restaurant managers [27] to understand the current trends and consumer segmentation that allow efficient targeting. In the favorable context of an increasing market for the hospitality industry in Romania, the need of such a study becomes “a must” for managers in order to obtain the above-mentioned advantages, since similar studies had not been yet conducted in Romania.

The results obtained suggest that the Romanian restaurant market in Cluj-Napoca is dominated by three segments of consumers, with the largest one being represented by “healthy-meal seekers”, which is a group of young women with medium and low incomes for whom the possibility of consuming healthy-meals within a restaurant is the most important factor during the decision-making process of restaurant selection. 

The present study has important managerial implications. On one hand, the empirical study emphasized the importance of understanding and identifying the main factors that consumers consider important in choosing a restaurant. Restaurant managers should admit that this process represents the starting point in designing a restaurant concept, and is fundamental information underlying management decisions. Moreover, the consumer segmentation is fundamental for building efficient marketing strategies and competitive advantages. According to the results obtained, managers should focus on extrinsic attributes such as cleanliness. On the other hand, the study offers important information regarding the consumer perception of food, which has recently changed significantly, especially regarding the segment of young consumers. Scholars consider “generation Y” to be the next most powerful segment in the restaurant industry due to their specific behavior and the ability to influence others, while being influenced by the internet and technological evolution. The finding that the “healthy-meal seekers” represents the largest segment of consumers has enormous implications for the “key players” [65] in the restaurant industry (public authorities and restaurant managers), which, according to Newson et al. [65], must cooperate to support and improve public health by direct and indirect measures. While public authorities have the instruments for contributing to the improvement of public health by introducing regulations [65,72] related to the nutritional information in the menu, and considering that in this context, customers are more likely to select healthy food items [73], the restaurant managers could introduce healthy food in their menus and adopt marketing strategies focused on increasing consumers’ awareness regarding healthy food [62]. It was observed also that providing nutritional information about meals increases the competitiveness of the restaurants and has a favorable impact on public health [74]. Introducing a calorie-labeling regulation in Romania could be considered as a viable option, by following the example of other countries where this is mandatory [75], but this should only be tackled after expanding the research area at the national level. Providing nutritional information about calories, macronutrients, and fat in restaurant menus might be of great help for consumers to make healthier eating choices [73], and support the segment that considers health to be the most important factor in the decision-making process of restaurant selection, as well as develop a competitive advantage. Applying this regulation must be preceded by a cost–benefit analysis. It was observed [76] that managers do have instruments to act toward a specific direction, which are often unutilized, by using a marketing mix focused on promotion strategies in order to drive the consumers’ attention toward healthy food choices. 

Future research directions could address other stakeholders interested in the restaurant market and its development. Also, a thorough analysis of consumer lifestyle, food preferences, and favorite dining places could be explored. The present study concluded that Romanian consumers prove to be health conscious when they select a restaurant. Thus, future strategies to increase public health could be easier to adopt because of the existent favorable perception of the population. Similar research could be conducted in the other large cities from Romania (Bucharest, Timisoara, Brasov, and Iasi) to obtain a more complex view of the process of restaurant selection. Due to objective limitations (time and resources), the present study relied on convenience sampling, but further research should use sampling techniques that allow the generalization of the results. Another limitation is that the level of education was not taken into account, which represents an objective for future research. Also, it was impossible to obtain information about restaurant customers, since there are no statistics regarding this aspect. 

## Figures and Tables

**Table 1:** Internal and external factors affecting restaurant selection.

Internal Features	External Features
Food quality [10,11,12,13,27,30,31,32]	Online reviews and restaurants’ ratings [16]
Quality of the service [15].	Nutritional concern [19]
Experience [17]	Dietary considerations [7,21]
Physical environment [35]	Lifestyle [8]
Hygiene [36]	Brand [25]

**Table 2:** Socio-demographic characteristics of the respondents.

Characteristics (*n* = 276)	Variables	%
Gender	Female	50.0
	Male	50.0
Age	18–25	39.9
	26–30	22.1
	31–40	10.1
	41–50	9.4
	Over 50	3.3
	n.a.	15.2
Marital status	Married	21.2
	Unmarried	78.8
Personal monthly income	Less than 320 euros	28.3
	320–445 euros	30.8
	445–555 euros	18.8
	556–665 euros	5.8
	Over 665 euros	12.7
	n.a.	3.6

**Table 3:** Principal component analysis (PCA) of the factors influencing restaurant selection.

Eigenvalue	Variance %	Component	Item	Factor Loading
6.776	39.86	Price-quality, service, and marketing α = 0.93	Rating	0.863
			Promotions	0.863
			Rapidity of preparation	0.857
			Reviews	0.804
			Interior design	0.803
			Product presentation	0.797
			Low price	0.771
2.918	17.17	Product quality and variety of the menu α = 0.87	Hearty meals	0.797
			High nutritional value	0.793
			Existence of traditional meals	0.738
			Fresh ingredients (not frozen)	0.706
			International meals	0.703
			Variety of the menu	0.700
1.249	7.35	Healthy meals α = 0.82	The menu contains low-calorie food	0.898
			The menu contains food that can be consumed during a diet	0.781
1.194	7.03	Appearance α = 0.54	Ambiance	0.798
			Clean spaces	0.679
Total variance %	71.4	α = 0.9		

**Table 4:** Importance of factors in the selection of restaurants.

Item (*n* = 276)	Mean	SD
**Price quality, service, and marketing**	3.44	0.158
Ratings	3.43	1.075
Promotions	3.33	1.078
Rapidity of preparation	3.70	1.173
Reviews	3.42	1.146
Interior design	3.28	1.098
Product presentation	3.62	1.117
Low price	3.31	1.047
**Product quality and variety of the menu**	3.65	0.388
Hearty meals	3.46	1.090
High nutritional value	3.43	1.078
Existence of traditional meals	3.47	1.158
Fresh ingredients (not frozen)	4.21	1.156
International meals	3.26	1.047
Variety of the menu	4.07	1.073
**Healthy meals**	3.54	0.015
The menu contains low-calorie meals	3.53	0.978
The menu contains meals that can be consumed during a diet	3.55	1.170
**Appearance**	4.35	0.368
Ambiance	4.09	1.058
Clean spaces	4.61	0.865

**Table 5:** Final cluster centers.

Factors	Cluster 1 (*n* = 124)	Cluster 2 (*n* = 43)	Cluster 3 (*n* = 109)	*F*-value	Significance
Price quality, service, and marketing	0.38650	−1.80729	0.27328	141.963	0.000 ***
Product quality and variety of the menu	0.21023	−0.50473	−0.04005	77.844	0.000 ***
Healthy meals	0.78889	0.01731	−0.90428	59.973	0.000 ***

*** *p* < 0.001.

**Table 6:** Mean of factors.

Factor	Cluster 1 (*n* = 124)	Cluster 2 (*n* = 43)	Cluster 3 (*n* = 109)	Kruskal–Wallis *χ*^2^ Statistic	*p*-Value
	Mean (SD)	Mean (SD)	Mean (SD)		
Price quality, service, and marketing	3.89 (0.561)	1.70 (0.495)	3.62 (0.558)	χ^2^ = 117.708 df = 2	0.000 ***
Product quality and variety of the menu	4.04 (0.633)	2.91 (1.062)	3.48 (0.762)	χ^2^ = 50.771 df = 2	0.000 ***
Healthy meals	4.37 (0.521)	3.14 (0.786)	2.74 (0.629)	χ^2^ = 177.219 df = 2	0.000 ***

*** *p* < 0.001.

**Table 7:** Demographic profile of consumers.

Characteristics	Variables	Cluster 1 (*n* = 124) ^a^	Cluster 2 (*n* = 43) ^a^	Cluster 3 (*n* = 109) ^a^	Chi-Square	*p*-Value
Gender	Female	69(56.6%)	27(62.8%)	41(37.6%)	χ^2^ = 11.6004 df = 2	0.003 **
	Male	53(43.4%)	16(37.2%)	68(62.4%)		
Age	18–25 years	58(54.72%)	10(24.39%)	42(48.28%)	χ^2^ = 37.2883 df = 8	0.000 ***
	26–30 years	21(19.81%)	16(31.71%)	27(31.03%)		
	31–40 years	18(16.98%)	6(14.63%)	4(4.60%)		
	41–50 years	2(1.89%)	10(24.39%)	14(16.09%)		
	Over 50 years	7(6.60%)	2(4.88%)	0(0.0%)		
Marital status	Married	22(17.7%)	18(41.9%)	18(16.8%)	χ^2^ = 13.1166 df = 2	0.001 **
	Unmarried	102(83.0%)	25(58.1%)	89(83.9%)		
Personal monthly income	Less than 320 euro	40(33.3%)	14(35.0%)	24(22.6%)	χ^2^ = 20.1948 df = 8	0.010 *
	320–445 euro	35(29.8%)	12(30.0%)	38(35.9%)		
	445–555 euro	22(18.3%)	14(35.0%)	16(15.1%)		
	556–665 euro	7(5.8%)	0(0.0%)	9(8.5%)		
	Over 665 euro	16(13.3%)	0(0.0%)	19(17.9%)		

a–Missing values excluded. * *p* < 0.05, ** *p* < 0.01, *** *p* < 0.001.

**Table 8:** Binary logistic regression.

Variables	Cluster 1				Cluster 2				Cluster 3			
	Odds Ratio	*p*-Value	95%CI		Odds Ratio	*p*-Value	95%CI		Odds Ratio	*p*-Value	95%CI	
			Lower	Higher			Lower	Higher			Lower	Higher
Gender												
Male ^a^	0.522	0.026 *	0.295	0.925	0.467 *	0.042	0.224	0.973	3.145	0.000 ***	1.739	5.686
Age												
26–30 years ^b^	0.544	0.073	0.279	1.059	3.324 *	0.011	1.318	8.381	0.974	0.939	0.496	1.911
31-40 years ^b^	1.678	0.258	0.684	4.115	3.621 *	0.028	1.148	11.417	0.212	0.009 **	0.066	0.684
41-50 years ^b^	0.075	0.001 ***	0.017	0.334	6.841 ***	0.000	2.412	19.393	1.826	0.188	0.745	4.475
>50 years ^b^	3.589	0.126	0.699	18.426	3.285	0.177	0.585	18.459				

^a^: Compared to female; ^b^: Compared to 18–25 years. * *p* < 0.05, ** *p* < 0.01, and *** *p* < 0.001.
